# The Host Range and Risk Assessment of the Stem-Boring Weevil, *Listronotus setosipennis* (Coleoptera: Curculionidae) Proposed for the Biological Control of *Parthenium hysterophorus* (Asteraceae) in Pakistan

**DOI:** 10.3390/insects12050463

**Published:** 2021-05-17

**Authors:** Philip Sebastian Richard Weyl, Abdul Rehman, Kazam Ali

**Affiliations:** 1 Centre for Agriculture and Biosciences International (CABI), Rue des Grillons 1, 2800 Delémont, Switzerland; 2 Centre for Agriculture and Biosciences International (CABI), Opposite 1-A, Data Gunj Baksh Road Satellite Town, Rawalpindi 43600, Pakistan; a.rehman@cabi.org (A.R.); k.ali@cabi.org (K.A.)

**Keywords:** classical biological control, invasive weed, exotic, weevil, stem miner, sunflower, risk analysis

## Abstract

**Simple Summary:**

Parthenium is an extremely damaging weed in Pakistan and there are not many sustainable and effective management options for its control. Biological control may offer a solution, provided that effective and safe agents are released. In this study we explore the host range and potential risk of a weevil, *Listronotus setosipennis*, in a Pakistani context. We tested several native and crop or ornamental species that have cultural importance in Pakistan. Our results suggest that the risk of releasing this weevil into Pakistan for the control of parthenium is extremely low and the benefits are likely to be great.

**Abstract:**

Parthenium, or *Parthenium hysterophorus*, has extended its range in Pakistan throughout Punjab and into Khyber Pakhtunkhwa, the Federally Administrated Tribal Areas, Azad Jammu and Kashmir, and Sindh Provinces. Without control measures against parthenium, the negative impacts of this weed will go unchecked having deleterious effects on native biodiversity, human and animal health, as well as crop productivity. The weevil *Listronotus setosipennis* was obtained and imported from the Plant Health and Protection of the Agricultural Research Council (ARC-PHP), in Cedara, South Africa, in April 2019. A total of 22 plant species or cultivars in the Asteraceae family were assessed during no-choice oviposition tests in Pakistan. During these tests, the only plant species accepted for oviposition were the 10 cultivars of *Helianthus annuus* that are grown in Pakistan. All cultivars were thus tested for development of *L. setosipennis* from egg to adult. Only three cultivars were able to support some larval development, but at such low levels that it is unlikely to be the basis of a viable population. To support this, a risk assessment was conducted to ascertain the probability of *L. setosipennis* being able to sustain viable populations in the field, the results of which concur with native (Argentina) and introduced (Australia) field host-range information where *L. setosipennis* has never been recorded as a pest of sunflowers. The results of laboratory-based host-range trials, together with host records from its native and introduced range, indicate that *L. setosipennis* is sufficiently specific to parthenium and is thus suitable for release in Pakistan.

## 1. Introduction

*Parthenium hysterophorus* L. (“parthenium”) is a highly invasive ruderal annual weed which is spreading rapidly through vast areas of the tropics and sub-tropics, with highly deleterious effects on native biodiversity, human and animal health, and productivity of both crops and grasslands [1]. Parthenium has an extensive native range in the Americas, occurring in the eastern USA as far north as New York and Ohio, throughout Mexico and Central America into South America, and extending as far as Argentina and Chile [2,3]. It was first recorded outside its native range in the 19th century (1810 in India, 1880 in South Africa), but most introductions date from the 1950s and 1960s. Parthenium has since then invaded 48 countries, as reported by Shabbir et al. [1]. In Pakistan, parthenium was first reported from Gujarat district of the Punjab Province in the 1980s [4]. For the first 20 years following its introduction it remained restricted to northern Punjab, but since 2000, its range has extended throughout Punjab and into Khyber Pakhtunkhwa (KP), the Federally Administrated Tribal Areas (FATA), Azad Jammu and Kashmir, and Sindh Provinces. It has spread along roads, canals and rivers, and the invasion has been further exacerbated by irrigation and floods [5]. Parthenium is rapidly spreading in various parts of Punjab, KP and Kashmir, and is becoming a dominant weed in different terrestrial ecosystems. The weed is highly invasive and is reported as a major weed for agro-ecosystems in Pakistan [6,7,8,9,10,11,12,13,14].

Despite the extent of parthenium and its deleterious effect on the livelihoods of millions of people globally, control programmes have not been given much attention. Successful management of parthenium is possible through a combination of control methods including biological and chemical control, containment strategies, the utilisation of competitive plant species and other cultural control methods [15,16,17]. Several herbicides have been shown to be effective in Pakistan [18,19], but research shows that continual follow-up treatments are required to remove new emerging plants and provide effective, long-term suppression of parthenium [20]. Financial constraints may, however, render chemical control unfeasible for many land owners. Hand-weeding is a labour intensive, although common, weed control practice in Africa and Asia [21], and now Pakistan, but it carries health risks (allergy/irritant) associated with frequent contact with parthenium. Subsistence farmers are particularly affected by parthenium invading grazing and arable land due to high levels of disturbance [22]. Reduction of grazing pressure by the lowering of livestock densities can be an effective means of managing parthenium [23], particularly after the establishment of biological control agents, but destocking may not be feasible in community and pastoral systems.

It is widely acknowledged that integrated control is the most effective strategy in managing pests. In the case of plants, it involves the use of herbicides, manual or mechanical control, and biological control agents in an integrated way. The main benefits of classical biological control are that the agents establish self-perpetuating populations and often establish throughout the range of the target weed, including areas which are not accessible for chemical or physical control; control of the weed is permanent; the cost is low relative to other approaches and usually requires a once-off investment; and benefits can be reaped by many stakeholders regardless of their financial status and whether they contributed to the initial research [24]. Pakistan does not have a long history of weed biological control, with only a single introduction of a single agent, *Cactoblastis cactorum* against *Opuntia* species [25].

In most cases, weed biological control relies on a classical biological control approach, in which natural enemies from the native range have been intentionally released as biological control agents in the introduced range after host range testing. In some cases, however, the agent has been found to be already present following an unknown or accidental introduction. In Pakistan, biological control options against parthenium are limited and there have so far been no deliberate introductions of agents to control parthenium. Nevertheless, *Zygogramma bicolorata* Pallister, known as the Mexican or parthenium leaf beetle, which was deliberately introduced from Mexico to India in 1983, was reported in Pakistan in 2006 and now has a widespread distribution in the country [26]. The inadvertent introduction of *Z. bicolorata* has offered Pakistan an opportunity to benefit from the biological control of parthenium with limited investment into host range testing and applications for permissions for release. Although future research into the biology, ecology and impact of this species in Pakistan is warranted, studies have shown that this species alone is unlikely to achieve full control of parthenium [27].

The winter rust, *Puccinia abrupta* Diet. & Holw. var. *partheniicola* (Jackson) Parmelee, has also been recorded from Pakistan and is now widely distributed throughout the range of parthenium in Pakistan [28,29]. Although the presence of this rust in Pakistan will aid in the management of parthenium, it is likely that its impact will be limited by abiotic factors. Temperature and dewpoint have been shown to be the limiting factors for urediniospore infection and survival, thus limiting impact of this rust in Australia where it was deliberately introduced [27]. Whilst it would be useful to monitor the distribution of this rust on an annual basis in winter, any redistribution would be unlikely to achieve a better level of control than is already experienced in the field [29].

Therefore, strategically, the resources available for biological control should focus on the evaluation of additional agents for release into Pakistan in order to obtain long-term sustainable control of parthenium [29]. The stem-boring weevil, *Listronotus setosipennis* (Hustache), another potential biological control agent for parthenium, is native to Brazil and Argentina. This weevil has had previous testing in Australia, South Africa and Ethiopia, which indicated that it is host specific and a safe biological control agent and has been released in these three countries as well as Uganda. With a view of evaluating *L. setosipennis* as a biological control agent for parthenium in Pakistan, CABI applied for the importation of this weevil to perform host range testing in its post entry quarantine facility in Rawalpindi [29]. The national quarantine authorities (Plant Sciences Division of the Pakistan Agricultural Research Council (PSD PARC) approved the importation of the weevil specifically for this purpose, and 200 adult *L. setosipennis* were imported from the rearing facility at the Plant Protection Research Institute of the Agricultural Research Council (ARC-PHP), in Cedara, South Africa, in April 2019 [29]. The import permit for *L. setosipennis* was issued by the Ministry of National Food Security and Research, Department of Plant Protection, Plant Quarantine Division under permit number IPKA-3787-016/18-2019. The aim of this study was to determine the host specificity of *L. setosipennis* in a Pakistani context.

## 2. Materials and Methods

### 2.1. Host-Range Testing

#### 2.1.1. Test Plant List Appropriate for Pakistan

It is generally accepted that species closely related to the target species are at greater risk of attack than species more distantly related. Therefore, test plant lists are established based on phylogenetic relationships between the target weed and other plant species [30]. The family Asteraceae is one of the most diverse and widespread families of vascular dicotyledonous plants, with species that are perennial, annual or biennial herbs, sub-shrubs and shrubs. The members of this family are abundantly present throughout the world except the Antarctica region [31]. The Asteraceae is a large family in Pakistan, which is represented by 758 species in 15 tribes. The tribe Heliantheae to which *Parthenium hysterophorus* belongs is only represented by 27 species in Pakistan. Comprehensive testing of the host range of *L. setosipennis* has already been done on a total of 151 species from 27 families, in Australia (73 species and/or cultivars), South Africa (52 species and/or cultivars) and Ethiopia tested (36 species and/or cultivars) (Table S1). In addition to this, a total of 39 species and/or cultivars within Helianthieae have already been tested in Australia, Ethiopia and South Africa. Since so many species have already been tested in a wide range of families and tribes, as well as specifically in Heliantheae, a condensed test plant list is justified for Pakistan (22 species and/or cultivars), focusing on native and crop or ornamental species that have overlapping distributions with parthenium and are likely to be at a higher risk of non-target attack (Table 1). During the process of the formation of the test plant list, advice and input was received from Dr. Amir Sultan, PSO, National Herbarium, NARC, Pakistan. The final test list contained representatives of eight tribes with both native and crop species (as well as ornamentals) of economic and cultural importance in Pakistan (Table 1). In this case, Pakistan has been able to greatly benefit from the work already done in other countries, namely Australia, South Africa and Ethiopia, which has significantly reduced the cost and time required for the evaluation of the biological control agent, *L. setosipennis.*

#### 2.1.2. Insect Cultures

Following importation from South Africa, the *L. setosipennis* culture was built up and maintained in quarantine at a day/night cycle of 14 L:10 D, mean temperature of 25 ± 5 °C, and mean relative humidity of 65 ± 5%. A culture of about 1000 adult weevils was maintained at all times. The nocturnal adult weevils feed mainly on parthenium leaves and flowers. The females oviposit in holes chewed in flowers, leaf bases or stem surfaces and axillary buds and seal the hole with black frass [32]. In the field in the native range, ten or more eggs were observed per plant, occasionally with two eggs per flower [33]. Oviposition can occur throughout the year as long as suitable plants are available [33]. Larvae emerge after 3–5 days. First instar larvae are highly mobile on the plant and feed in the flowers while the mature larvae feed within the stem and move to the root where they feed prior to pupation. Emergence of adults from pupae is triggered by an increase in soil moisture [33]. Adults diapause in the soil during the dry season [34]. The life cycle takes about 7 weeks and adult weevils can live up to 8 months [33].

#### 2.1.3. General Considerations for Host Range Trials

In all tests, potted plants (not excised leaves or stems) were used to ensure that test conditions were as optimal as possible. Depending on the size of the plant, the appropriate pot size was used, ranging from 1–4 L pots, filled with a standard commercial potting soil. Standardized potting soil was prepared by mixing 18 kg of all-purpose potting soil (Miracle-Gro) with 185 g Osmocote fertilizer with N:P:K of 19-6-12 and 125 g Dolomite lime. The plants were watered ad libitum. Each test plant was ensured to be in the correct phenological stage for oviposition, which was flowering with still-developing florets.

Host-range trials for *L. setosipennis* were conducted by comparing oviposition on parthenium to that on the 22 non-target plant species (including the 10 sunflower cultivars). This was achieved through an initial series of no-choice oviposition tests for all 22 selected non-target species (including all 10 sunflower cultivars), followed by an assessment of larval survival on non-target plants that proved suitable for oviposition in no-choice tests, and finally, the development of a risk assessment for non-target species that received eggs in no-choice tests.

No-choice tests were conducted as the initial step to determine whether any non-target species would be accepted for oviposition in the absence of parthenium. While no-choice tests determine the plant species on which the insect is physiologically capable of feeding and egg laying, i.e., the ‘physiological’ host range [35], choice tests provide a more realistic view of those species the insect may feed on under natural conditions i.e., the ‘realized’ host range [35,36]. Unfortunately, under quarantine conditions at the CABI post entry quarantine facility in Rawalpindi, multiple choice tests were not possible due to space limitations and thus the data presented in this study are conservative and likely exaggerate the potential risk.

#### 2.1.4. No-Choice Oviposition Tests

Adults used in no-choice oviposition tests were between two and four weeks old, to ensure that females were of egg-laying age following the period of pre-oviposition while ovarioles develop. For each test series of no-choice oviposition tests, about 100 adults from the culture maintained in quarantine were placed in several Petri dishes containing filter paper moistened with a dilute (2%) sodium hypochlorite solution. Adults were held for up to 24 h, depending on the duration required to collect sufficient mating pairs. Copulating weevils were collected to ensure that they were male and female as it was difficult to differentiate the sexes morphologically.

Five pairs of adults were exposed to a single test or parthenium control plant at the correct phenological stage, in this case flowering, in separate gauze cages with mesh diameter of 1 mm^2^ (SE-1836) of dimensions 45 × 45 × 90 cm^3^ for five days, with a day/night cycle of 14 L:10 D, mean temperature of 25 ± 5 °C, and mean relative humidity of 65 ± 5%. For each series of tests, there was at least a single parthenium control plant. All plants were washed with water to remove any pests before the start of the trial, and watered daily during the trial. As eggs are difficult to accurately count without destructive sampling, egg counts were conducted only at the end of each experiment. After five days, all adults were removed from plants and returned to the culture but were not re-used in tests. All above-ground parts of each test and control plant were carefully examined for eggs, using a microscope, and plants were dissected. Between four and five replicates were conducted for each plant species tested and 25 control plants were used in total. As data were not normally distributed, even if log-transformed, non-parametric independent samples Kruskal–Wallis test was conducted by comparing the number of eggs laid on test plant replicates against that on parthenium control plants. Data were analysed using IBM SPSS Statistics v25.

#### 2.1.5. Larval Development Tests

Plants that received eggs in no-choice tests were further tested in larval development tests; in this case only the sunflower varieties were tested, because they were the only ones accepted for oviposition. In order to obtain eggs for the trial, adult weevils from the culture were exposed to severed parthenium flowers on moistened filter paper in Petri dishes, for a period of 24 h. Petri dishes with eggs in flowers were then held for one to two days. The rim of the Petri dish was sealed using a layer of Parafilm to avoid desiccation.

In South Africa, several attempts using eggs removed from flowers and placing newly hatched larvae in varying numbers on plants, failed to achieve adequate larval survival on parthenium [37]. Therefore, following the same techniques as Strathie and McConnachie [37], parthenium flowers containing eggs were used for larval development tests. The flowers, each containing single one- or two-day old eggs, were tied together with cotton thread in clusters of about six flowers each, and attached at five different points along the stems of parthenium and sunflowers, with a total of 30 eggs per plant, and kept in separate gauze cages (SE-1872) of dimensions 45 × 45 × 180 cm^3^ for 11 weeks. Four replicates were conducted for each test plant cultivar as well as eight replicates for parthenium. After three weeks, plants were checked several times per week for adult emergence, and the newly emerged adults were recorded. New flowers and dead leaves were removed from plants periodically so that adult weevils were easier to find. After about 11 weeks all plants were dissected and the number of larvae and pupae were recorded. Pupae were transferred to moistened soil on filter paper within Petri dishes. Non-parametric independent samples Kruskal–Wallis test was conducted to compare the number of adults that developed on each sunflower cultivar with those on the parthenium control plants. Data were analysed using IBM SPSS Statistics v25 (SPSS Inc., Chicago, IL, USA).

### 2.2. Risk Assessment

A risk assessment, as performed by Wan and Harris [38], was conducted to ascertain and quantify the risks to non-target species, in this case, sunflower cultivars, that supported oviposition, with or without complete development from egg to adult. This assessment quantified the suitability of several sunflower cultivars as hosts to sustain viable populations of *L. setosipennis*. The performance of *L. setosipennis* at various stages (oviposition and development) of the host selection process on sunflower cultivars was compared as a proportion of that on parthenium.

The performance criteria used included relative oviposition preference (R1) and relative performance from egg to adult (R2). For each criterion, R represented the weevil’s performance on the test plant proportional to that on parthenium in the same test. Oviposition preference was determined by the mean number of eggs laid per plant for each test plant species during no-choice tests. Relative performance was determined by the number of individuals that completed development from egg to adult on test plant species during larval development tests relative to that on parthenium. The reproductive risk (i.e., the probability of producing a viable population) was calculated as the product of oviposition preference and survival from egg to adult (R1 × R2). Zero values were designated as 0.001 to facilitate calculations, as used by others [38,39,40].

## 3. Results

### 3.1. No-Choice Oviposition Tests

In the no-choice tests, adult feeding was sporadic and difficult to quantify (or even find in some cases) and thus was deemed negligible and not recorded. In the no-choice oviposition tests, all 10 sunflower (*H. annuus*) cultivars that were assessed received eggs by *L. setosipennis* females, while all other test plants were completely avoided and no eggs were recorded (Table 1). Average (±SE) egg numbers on sunflowers ranged from 5.40 ± 1.9 (cultivar FMC-2) and 5.40 ± 1.7 cultivar S278 to 20.6 ± 6.3 on cultivar ESNH-013, compared to 185.5 ± 15.2 on parthenium as the control. The difference in numbers of eggs oviposited on the sunflower test cultivars compared to the control plants was highly significant (Kruskal–Wallis, H = 52.8; df = 12; *p* < 0.001).

### 3.2. Larval Development Tests

The ten sunflower cultivars that received eggs in no-choice tests were assessed in larval development tests, to determine whether these plants could support complete development. Development was only recorded on three sunflower cultivars (four individual plants) (ParSun-3, S-278, Hysun-33), and a total of only 6 adults emerged from an initial total of 360 eggs placed on these 3 sunflower cultivars (single adult on ParSun-3, while 2 adults on one replicate of cultivar Hysun-33 and finally 2 replicates of S-278 supported development of 1 and 2 adults each). This translates to 0.8 % survival from egg to adult on ParSun-3, 1.6 % survival from egg to adult on Hysun-33 and 2.5 % survival from egg to adult on S-278, while, on parthenium there was 42.9% survival from egg to adult. This is significantly fewer adults in comparison, to the successful development recorded on the parthenium plants setup with a total of 103 adults from 240 eggs (Kruskal–Wallis, H = 37.777; df = 10; *p* < 0.0001) (Table 2).

### 3.3. Risk Assessment

Oviposition under no-choice conditions and survival from egg to adult were utilised to quantify the potential reproductive risk, i.e., the likelihood of these sunflower cultivars being able to sustain a viable population of *L. setosipennis*. Although during larval development tests, no larvae completed development on seven of the ten sunflower cultivars (Table 2); these cultivars were included in the risk assessment as they had received eggs during the no-choice tests, and as expected these resulted in negligible final risk assessment scores (Table 3). The likelihood of *L. setosipennis* being able to sustain viable field populations on the three sunflower cultivars (ParSun-3, Hysun-33, S-278) on which one, two and three individuals respectively completed development in larval development trials, was also extremely low, as indicated by their final risk assessment scores (1.28 × 10^−3^ for S278, 1.22 × 10^−3^ for ParSun-3 and 8.76 × 10^−4^ for Hysun-3) which were more than three orders of magnitude less than that of parthenium (Table 3). The combination of very low incidence of oviposition and very low ability to complete development to the adult stage strongly indicates an inability to sustain a viable population on these three sunflower cultivars.

## 4. Discussion

It is imperative that the spread and impact of parthenium in Pakistan be contained as it causes serious agricultural, environmental, and human and animal health problems, resulting in severe economic losses. The cost of herbicide applications, with further repeated, seasonal, follow-up applications required to achieve maintenance levels in the long-term is extremely expensive. Chemical control although effective in Pakistan [19], requires successive follow-up applications that are applied consistently and at the correct frequency or else, parthenium infestations revert to their original densities prior to the implementation of chemical control efforts [20]. The use of *L. setosipennis* as a biological control agent is therefore likely to be particularly beneficial for areas with communities with limited resources for chemical control, as well as in areas of agricultural production and conservation significance, with the beneficial attributes of the agent compounded with time, ultimately reducing the need for herbicide use.

In Australia where various management strategies (including biological control, chemical control, pasture management, competitive plant species, and vehicle wash-down facilities) have been implemented for several decades, there has been a measurable reduction in the losses caused by the weed [16,34]. Biological control using a combination of natural enemies is recognised as one of the major management strategies required, as it has been proven to reduce the abundance and impact of parthenium in most situations and seasons, with significant economic benefits, although serious infestations of the weed can still occur in some seasons and areas [34]. Reductions in parthenium populations using nine natural enemies have been achieved in Australia since the research programme was initiated in 1977 [34]. If other countries are to have any hope of achieving similar levels of control, they will need to follow this model of multiple agents. In Pakistan, two biological control agents are present, *Z. bicolorata* and *P. abrupta* var. *partheniicola*; however, there is a need for additional introductions of effective, host-specific insect agents, such as *L. setosipennis*, to curb the spread and impact of the weed.

During host-specificity testing for the Australian research programme, 68 plant species from 26 families were tested; for the South African research programme, 39 plant species from 2 families were tested; and for the Ethiopian research programme, 31 plant species from 7 families were investigated (Table S1). Limited oviposition was recorded on *Zinnia* and *Helianthus annuus* cultivars in no choice tests conducted in South Africa [37], Brazil and Australia [33], while Ethiopian tests had no non-target oviposition [41]. In quarantine multiple-choice cage tests with adults in Australia, there was no feeding or oviposition on any of the test plants, but, between 19 and 90 eggs were laid on the parthenium plants in each test [33]. In South Africa there was limited oviposition (between 1 and 10 eggs) on eight cultivars of sunflower, *H. annuus* under quarantine choice cage conditions [37]. This is not unusual under cage conditions in quarantine and the further risk assessment considering larval development resulted in extremely low risk of *L. setosipennis* being able to sustain a population on sunflowers. Guided by these results, *L. setosipennis* was shown to be safe for release in all three countries and first released in 1982 in Australia, 2013 in South Africa and 2016 in Ethiopia. To complement the existing knowledge of the host range of *L. setosipennis* established from tests conducted in Brazil, Australia, South Africa and Ethiopia, additional host-range testing was conducted in a Pakistani context.

Of the 13 plant species in the Asteraceae family that were assessed during no-choice oviposition tests in Pakistan, 12 were not accepted for oviposition by *L. setosipennis*, indicating a narrow physiological host range. These results are in line with other host range tests already conducted in Australia [33], South Africa [37] and Ethiopia [41]. Of all the species tested, only the sunflower cultivars were selected for oviposition by *L. setosipennis*, albeit receiving an extremely limited number of eggs. Typically, these would then be tested under multiple choice conditions to obtain an understanding of the realized host range; however, due to space limitations in the quarantine facility in Pakistan this was not possible and thus the data presented in this study are conservative and likely over-inflate the risk. Despite this, complete development of *L. setosipennis*, even though extremely limited, could be achieved on three sunflower cultivars (ParSun-3, Hysun-33, S-278) under laboratory conditions. However, quantified analyses, combining the relative oviposition performance under no-choice conditions and relative performance from egg to adult, indicated that the likelihood of *L. setosipennis* being able to sustain viable populations or have an impact on these three cultivars in the field was extremely low (1.28 × 10^−3^ for S278, 1.22 × 10^−3^ for ParSun-3 and 8.76 × 10^−4^ for Hysun-3) and negligible relative to parthenium. Although it is not possible to completely exclude the risk of potential spillover of adults onto sunflowers if parthenium is growing in close proximity, our analysis suggests that this is unlikely and even more unlikely to cause any considerable damage to sunflowers.

Similar to the current study, in both Australia and South Africa, *L. setosipennis* oviposited on some sunflower cultivars under no-choice and paired-choice conditions, and complete development occurred in some instances [33,37]. It was concluded in Australia that oviposition by *L. setosipennis* adults confined to sunflowers in the laboratory was less than 1% of that on parthenium, and less than 2% of those eggs survived to adulthood [33]. Prior to research by Wild et al. [33], there were no known host records for *L. setosipennis*. It had not been recorded as a pest of any crop or pasture species in South America and was not known to entomologists of the National Institute of Agricultural Technology in Argentina [33]. Sunflowers are native to the Americas and widely cultivated in northern Argentina, but *L. setosipennis* had never been recorded attacking the crop [40]. Additionally, Queensland Government entomologists in South America conducted extensive searches between 1975 and 1990 on the closely related plants in the *Ambrosia* and *Xanthium* species but never found *L. setosipennis* larvae in these plants [33].

These results are corroborated by the fact that almost four decades after its establishment in Australia, *L. setosipennis* has never been recorded feeding on sunflowers, nor is it a recorded pest of sunflowers in its native range of Argentina where sunflowers are also widely cultivated [42]. During surveys on related plants growing in and among parthenium infestations in Queensland, Australia, while evaluating biocontrol agents in the field from 1978 until 2003, parthenium-infested areas were regularly visited and consultations were held with farmers growing crops, including sunflowers, in the parthenium invaded areas of Central Queensland. *Listronotus setosipennis* was widespread and seasonally abundant throughout the parthenium infestations of North and Central Queensland but there were never any reports of damage to sunflowers (R. McFadyen, pers. comm., cited in [37]). In contrast, damage to parthenium was sometimes very significant, especially to the first generation of young plants that germinated after spring rains, when almost all parthenium plants over an area of several hectares were destroyed by *L. setosipennis* (R. McFadyen, pers. comm., cited in [37]). Similarly, in South Africa, a risk assessment (equivalent to the one conducted in the current study) concluded that it would be impossible to sustain field populations on the cultivars assessed and that the risk of viable field populations being sustained on two susceptible *H. annuus* cultivars was extremely low (0.012% and 0.048% respectively) [37].

## 5. Conclusions

The only known host of *L. setosipennis* in its native range of Argentina is parthenium [34]. The Australian research programme assessed 68 non-target plant species, including 18 members of the Asteraceae (including six sunflower cultivars) as well as commercially cultivated members from another 25 families [33]. In South Africa, *L. setosipennis* was tested on 38 native and economically important non-target Asteraceae species [32,37], while in Ethiopia it was evaluated on 31 plant species [41]. All countries concluded that *L. setosipennis* is host specific and a safe biological control agent for use against parthenium, leading to approval for release by all regulatory authorities after a thorough review of submitted risk assessments. The quarantine laboratory assessments of the host range of *L. setosipennis* conducted in Pakistan are in line with these findings and in combination with evidence of the weevil’s native field host range in South America and introduced field host range in Australia, South Africa and Ethiopia indicate that it is highly unlikely that *L. setosipennis* will cause any damage to any plant, native or cultivated, other than parthenium, in Pakistan.

## Figures and Tables

**Table 1:** Prioritised native and economically and culturally important Asteraceae plant species in Pakistan tested with *Listronotus setosipennis* in no-choice oviposition tests, between 2019–2021.

FAMILY	Common Name	No. Valid Replicates	No. of Eggs Mean ± SE	Relative Preference
Tribe				
*Species*				
ASTERACEAE				
** Heliantheae**				
*Parthenium hysterophorus*	Gajar booti	25	185.5 ± 15.2	1.00
*Cosmos bipinnatus ^C^*	Cosmos	4	0 ± 0	0
*Eclipta prostrata ^N^*	Bhangra weed	4	0 ± 0	0
*Helianthus annuus* (S278) ^C^	Sunflower	5	5.4 ± 1.7	0.03
*Helianthus annuus* (ParSun-3) ^C^	Sunflower	5	16.2 ± 5.3	0.09
*Helianthus annuus* (SF-0054) ^C^	Sunflower	5	9.6 ± 2.7	0.05
*Helianthus annuus* (KQS-FSH-1) ^C^	Sunflower	5	9.8 ± 5.0	0.05
*Helianthus annuus* (S-3950) ^C^	Sunflower	5	18.0 ± 7.9	0.10
*Helianthus annuus* (FMC-2) ^C^	Sunflower	5	5.4 ± 2.0	0.03
*Helianthus annuus* (S-2216) ^C^	Sunflower	5	12.8 ± 5.7	0.07
*Helianthus annuus* (ESNH-013) ^C^	Sunflower	5	20.6 ± 6.3	0.11
*Helianthus annuus* (HySun-33) ^C^	Sunflower	5	5.6 ± 4.5	0.03
*Helianthus annuus* (SX-4045) ^C^	Sunflower	5	5.6 ± 3.7	0.03
*Rudbeckia laciniata* ^C^	Black-eyed Susan	4	0 ± 0	0
*Zinnia elegans* ^C^	Zinnia	4	0 ± 0	0
** Anthemideae**				
*Dendranthema indica* ^C^	Gul-e-Daudi	4	0 ± 0	0
** Astereae**				
*Callistephus chinensis* ^C^	Aster	4	0 ± 0	0
** Calenduleae**				
*Calendula officinalis* ^C^	Pot marigold	4	0 ± 0	0
** Cicorieae**				
*Lactuca sativa* ^C^	Lettuce	4	0 ± 0	0
** Coreopsideae**				
*Dahlia pinnata* ^C^	Dahlia	4	0 ± 0	0
*Bidens bipinnata ^N^*	Bidens	4	0 ± 0	0
** Cynareae**				
*Carthamus tinctorius* ^C^	Safflower	4	0 ± 0	0
** Tageteae**				
*Tagetes erecta* ^C^	Gul-e-Ashrafii	4	0 ± 0	0

^C^ indicates crop species or cultivars in Pakistan. ^N^ indicates native species in Pakistan.

**Table 2:** *Listronotus setosipennis* larval development (from 30 eggs per replicate) on sunflower cultivars that received eggs in no-choice tests.

Plant Species	No. of Adults Emerged Mean ± SE	No. of Pupae Mean ± SE	No. of Live Larvae Mean ± SE	Relative Performance
*Parthenium hysterophorus*	12.88 ± 1.56	0.50 ± 0.27	3.63 ± 0.75	1
*Helianthus annuus* (S278)	0.75 ± 0.48	0	0	0.044
*Helianthus annuus* (ParSun-3)	0.25 ± 0.25	0	0	0.014
*Helianthus annuus* (SF-0054)	0	0	0	0
*Helianthus annuus* (KQS-FSH-1)	0	0	0	0
*Helianthus annuus* (S-3950)	0	0	0	0
*Helianthus annuus* (FMC-2)	0	0	0	0
*Helianthus annuus* (S-2216)	0	0	0	0
*Helianthus annuus* (ESNH-013)	0	0	0	0
*Helianthus annuus* (HySun-33)	0.50 ± 0.50	0	0	0.029
*Helianthus annuus* (SX-4045)	0	0	0	0

**Table 3:** Risk assessment of the performance of *Listronotus setosipennis* on sunflower cultivars on which oviposition and complete development occurred during no-choice and larval development tests, relative to *Parthenium hysterophorus*.

Cultivar	Oviposition Preference ^1,2,3^ (R1)	Performance ^1,2,4^ (R2)	Reproductive Risk (R1 × R2)
*Parthenium hysterophorus*	1.00	1.00	1.00
*Helianthus annuus* (S278)	0.03	0.044	1.28 × 10^−3^
*Helianthus annuus* (ParSun-3)	0.09	0.014	1.22 × 10^−3^
*Helianthus annuus* (SF-0054)	0.05	**0.001**	5.18 × 10^−5^
*Helianthus annuus* (KQS-FSH-1)	0.05	**0.001**	5.28 × 10^−5^
*Helianthus annuus* (S-3950)	0.10	**0.001**	9.70 × 10^−5^
*Helianthus annuus* (FMC-2)	0.03	**0.001**	2.91 × 10^−5^
*Helianthus annuus* (S-2216)	0.07	**0.001**	6.90 × 10^−5^
*Helianthus annuus* (ESNH-013)	0.11	**0.001**	1.11 × 10^−4^
*Helianthus annuus* (HySun-33)	0.03	0.029	8.76 × 10^−4^
*Helianthus annuus* (SX-4045)	0.03	**0.001**	3.06 × 10^−5^

^1^ Performance values are proportional to *P. hysterophorus*. ^2^ Zero values were designated **0.001** to facilitate calculations. ^3^ Oviposition preference during no-choice tests (Table 1). ^4^ Complete development from egg to adult during larval development tests (Table 2).

## Data Availability

The data presented in this study are available on request from the corresponding author.

## Supplementary Materials

The following are available online at https://www.mdpi.com/article/10.3390/insects12050463/s1, Table S1: The test plant species from Australia, South Africa and Ethiopia that have already been tested with *Listronotus setosipennis* in each respective country prior to release. Each country found no evidence for the potential of *L. setosipennis* to have non-target impacts on either native or economically important crop species.
