# Correction: Weyl et al. The Host Range of the Stem-Boring Weevil, *Listronotus setosipennis* (Coleoptera: Curculionidae) Proposed for the Biological Control of *Parthenium hysterophorus* (Asteraceae) in Pakistan. *Insects* 2021, *12*, 463

**DOI:** 10.3390/insects12090763

**Published:** 2021-08-25

**Authors:** Philip Sebastian Richard Weyl, Abdul Rehman, Kazam Ali

**Affiliations:** 1 Centre for Agriculture and Biosciences International (CABI), Rue des Grillons 1, 2800 Delémont, Switzerland; 2 Centre for Agriculture and Biosciences International (CABI), Opposite 1-A, Data Gunj Baksh Road Satellite Town, Rawalpindi 43600, Pakistan; a.rehman@cabi.org (A.R.); k.ali@cabi.org (K.A.)

The authors would like to make the following corrections to this paper [1] following concerns due to breach of intellectual property and copyright, as well as significant overlap with an unpublished report. Consequently, the authors would like to make the following changes.

## 1. Title Correction

Given the changes required, the title required a slight change. The correct title of the article is “The Host Range of the Stem-Boring Weevil, *Listronotus setosipennis* (Coleoptera: Curculionidae) Proposed for the Biological Control of *Parthenium hysterophorus* (Asteraceae) in Pakistan”. We apologize for this error and state that the scientific conclusions are unaffected. The original article has been updated.

## 2. Simple Summary

In the article the removal of the risk analysis was required (Sections 2.2 and 3.3). To reflect this change, the third sentence of the simple summary required the deletion of ‘and potential risk’.

## 3. Abstract

In the article the removal of the risk analysis was required (Sections 2.2 and 3.3), as well as the contribution of the ARC-PHP to the study. To reflect this change, the sentence ‘To support this, a risk assessment was conducted to ascertain the probability of *L. setosipennis* being able to sustain viable populations in the field,’ has been deleted. In addition to this, ‘and South Africa was added to the sentence A total of 22 plant species or cultivars in the Asteraceae family were assessed during no-choice oviposition tests in Pakistan and South Africa’.

## 4. Introduction

Due to overlap with an unpublished report, the second paragraph of the introduction needed to be deleted completely.

A correction to the name of ARC-PHP was made in the last paragraph of the introduction. It now reads:

The national quarantine authorities (Plant Sciences Division of the Pakistan Agricultural Research Council (PSD PARC)) approved the importation of the weevil specifically for this purpose, and adult *L. setosipennis* were imported from the rearing facility of the Plant Health and Protection of the Agricultural Research Council (ARC-PHP), at Cedara, South Africa, in April 2019 [20]. The import permit for *L. setosipennis* was issued by the Ministry of National Food Security and Research, Department of Plant Protection, Plant Quarantine Division under permit number IPKA-3787-016/18-2019. The aim of this study was to determine the host specificity of *L. setosipennis* in a Pakistani context.

## 5. Materials and Methods

In Section 2.1.1, reference to data on the number of plant species tested in South Africa needed to be deleted, as these are in an unpublished report and the contribution of the ARC-PHP was acknowledged, corrected sentences:

Comprehensive testing of the host range of *L. setosipennis* has already been done in several countries including Australia [21], Ethiopia, and South Africa [22] (Table S1). In addition to this, a total of 19 species and/or cultivars within Heliantheae have already been tested in Australia and Ethiopia.

And:

In this case, Pakistan has been able to greatly benefit from the guidance of the ARC-PHP, South Africa, as well as work already done in other countries, namely Australia and Ethiopia, which has significantly reduced the cost and time required for the evaluation of the biological control agent *L. setosipennis.*

Sections 2.1.2–2.1.5 required changes due to overlap with an unpublished report, corrected paragraph:

2.1.2. Insect Cultures

Following importation from the ARC-PHP, Cedara, South Africa, the *L. setosipennis* culture was built up and maintained in the Pakistan quarantine at a day/night cycle of 14 L:10 D, mean temperature of 25 ± 5 °C, and mean relative humidity of 65 ± 5%. Due to space limitations, it was only possible to maintain a culture of approximately 1000 adult weevils. For detailed biology of this weevil, refer to [21].

2.1.3. General Considerations for Host Range Trials

In all tests, potted plants (not excised leaves or stems) were used to ensure that test conditions were as optimal as possible. Depending on the size of the plant, the appropriate pot size was used, ranging from 1–4 L pots, filled with a standard commercial potting soil. Standardized potting soil was prepared by mixing 18 kg of all-purpose potting soil (Miracle-Gro) with 185 g Osmocote fertilizer with N:P:K of 19-6-12 and 125 g Dolomite lime. The plants were watered ad libitum. Each test plant was ensured to be in the correct phenological stage for oviposition, which was flowering with still-developing florets.

We followed a typical host range testing procedure, following recommendations by [24], to assess the physiological and ecological host range. The physiological host range encompasses all plant species on which the insect, in this case *L. setosipennis*, can develop under no-choice conditions [26], while the ecological host range includes plant species which are utilized under natural conditions [26]. The testing sequence progressively reduces the degree of restriction, deleting unattacked plants at each stage, until only a few remain to be tested under conditions as natural as possible. This has proven to be a reliable way to determine the safety of potential biological control agents. Unfortunately, under quarantine conditions at the CABI post entry quarantine facility in Rawalpindi, multiple-choice tests were not possible due to space limitations, and thus the data presented in this study are conservative and likely exaggerate the potential risk.

2.1.4. No-Choice Oviposition Tests

Following guidance by ARC-PHP, South Africa, and previous studies [22], five pairs of adults were exposed to a single test or parthenium control plant at the correct phenological stage, in this case flowering, in individual fine mesh cages with mesh diameter of 1 mm^2^ (SE-1836) of dimensions 45 × 45 × 90 cm for five days, with a day/night cycle of 14 L:10 D, mean temperature of 25 ± 5 °C, and mean relative humidity of 65 ± 5%. Typical in host range testing, for each experimental setup, there was also a control plant. The no-choice tests were run in four consecutive series of experiments between 2019 and 2020.

2.1.5. Larval Development Tests

During the no-choice tests, oviposition was recorded only on the 10 sunflower cultivars and not on any of the other plant species tested. However, since sunflower is an important crop in Pakistan and oviposition has also been recorded on this species in Australia [21] and South Africa [27], it was considered a critical species. Due to space constraints in quarantine, in Pakistan, efforts were focused on larval development tests to determine whether this weevil would be able to sustain a viable population and thus cause damage to sunflower. Ten cultivars commonly grown in Pakistan (Table 2) were propagated in pots, and a total of four replicates for each were conducted, as well as for eight control plants.

A total of 30 eggs were placed on each sunflower plant, as well as the control plant, and each plant was kept in individual fine mesh cages (SE-1872) of dimensions 45 × 45 × 180 cm for 11 weeks. Each cage was inspected regularly for the emergence of adults, and after about 11 weeks, all plants were dissected, and the number of larvae and pupae were recorded. To test whether there is a difference between the number of adults emerging from parthenium compared to the sunflower cultivars, the non-parametric independent samples Kruskal–Wallis test was conducted. In addition to this, the same test was run excluding the control plant to determine whether there is a difference between the likelihood of any sunflower cultivars supporting the development of *L. setosipennis*. Data were analysed using IBM SPSS Statistics v25 (SPSS Inc., Chicago, IL, USA).

Section 2.2 was deleted completely due to overlap with an unpublished report.

## 6. Results

Section 3.2 required changes due to overlap with an unpublished report and to reflect changes made in the materials and methods, corrected paragraph:

*3.2. Larval Development Tests*

The ten sunflower cultivars that are considered important in Pakistan were tested in larval development tests. Development was only recorded on three sunflower cultivars (four individual plants) (ParSun-3, S-278, Hysun-33), and only six adults emerged from a total of 1200 eggs exposed to sunflower plants (single adult on ParSun-3, while two adults on one replicate of cultivar Hysun-33, and finally two replicates of S-278 supported development of one and two adults each). When only considering the three cultivars where development occurred, survival from egg to adult is 2.5% or less, and if all cultivars are considered, the likelihood of survival is 0.5%. In contrast, on their usual host, parthenium, there was a much greater survival from egg to adult of over 40%. There were significantly fewer adults on the sunflower cultivars in comparison to the successful development recorded on the parthenium plants setup, with a total of 103 adults from 240 eggs (Kruskal–Wallis, H = 37.777; df = 10; *p* < 0.0001) (Table 2). When the data from the control plants were excluded from the analyses, there was no difference in the likelihood of any sunflower cultivar supporting development to adult, suggesting that no one cultivar is more at risk, but rather that all cultivars are at low risk (Kruskal–Wallis, H = 11.897; df = 9; *p* < 0.219). The very low ability to complete development to the adult stage strongly indicates an inability to sustain a viable population on these sunflower cultivars.

Section 3.3, as well as Table 3, was completely deleted.

Table 1. Caption requires changes as well as the removal of the relative preference column since the risk analysis was removed this is irrelevant. Additionally a footnote was added acknowledging the contribution of ARC-PHP. New table is as follows:

Table 2. Caption requires changes as well as the removal of the relative preference column since the risk analysis was removed, as this is now irrelevant due to deletion of the risk analysis. New table is as follows:

**Table 2:** Development of *Listronotus setosipennis* larvae arising from 30 eggs per replicate on sunflower cultivars that are considered important in Pakistan.

Plant Species	No. Valid Replicates	No. of Adults Emerged Mean ± SE	No. of Pupae Mean ± SE	No. of Live Larvae Mean ± SE
*Parthenium hysterophorus*	8	12.88 ± 1.56	0.50 ± 0.27	3.63 ± 0.75
*Helianthus annuus* (S278)	4	0.75 ± 0.48	0	0
*Helianthus annuus* (ParSun-3)	4	0.25 ± 0.25	0	0
*Helianthus annuus* (SF-0054)	4	0	0	0
*Helianthus annuus* (KQS-FSH-1)	4	0	0	0
*Helianthus annuus* (S-3950)	4	0	0	0
*Helianthus annuus* (FMC-2)	4	0	0	0
*Helianthus annuus* (S-2216)	4	0	0	0
*Helianthus annuus* (ESNH-013)	4	0	0	0
*Helianthus annuus* (HySun-33)	4	0.50 ± 0.50	0	0
*Helianthus annuus* (SX-4045)	4	0	0	0

## 7. Discussion

To reflect changes in the results as well as the deletion of the risk analysis section the discussion required significant reworking, corrected discussion:

The results from the current study on the host range of *L. setosipennis* in a Pakistani context suggest that it is a safe biological control agent for release. Of the 22 Asteraceae plant species that were tested under no-choice conditions, oviposition by *L. setosipennis* was recorded only on the sunflower cultivars tested, suggesting a narrow physiological host range. These results are in line with other host range tests already conducted in Australia [21] and Ethiopia [22], where 68 plant species from 26 families and 31 plant species from 7 families were investigated, respectively. Limited oviposition was recorded on *Zinnia* and *Helianthus annuus* cultivars in no-choice tests conducted in Brazil and Australia [21], while Ethiopian tests had no non-target oviposition [22]. In quarantine multiple-choice cage tests with adults in Australia, there was no feeding or oviposition on any of the test plants, but between 19 and 90 eggs were laid on the parthenium plants in each test [21]. This is not unusual under cage conditions in quarantine, and further assessment of larval development indicated extremely low risk of *L. setosipennis* being able to sustain a population on sunflowers. Guided by these results, *L. setosipennis* was shown to be safe for release in three countries and first released in 1982 in Australia, 2013 in South Africa, 2016 in Ethiopia, and 2018 in Uganda [23].

Given that sunflower is an important crop worldwide, typically, for testing in Pakistan, sunflower should have been tested under multiple-choice conditions to obtain an understanding of the realized host range; however, due to space limitations in the quarantine facility in Pakistan, this was not possible, so efforts were focused on only conducting larval development tests. Thus, the data presented in this study are conservative and likely over-inflate the risk. Despite this, there was only minor survival and development recorded on three cultivars, namely ParSun-3, S-278, and Hysun-33; however, when comparing between cultivars, the number of adults does not suggest that one is more at risk than another, but rather that all varieties are at low risk. Given that the probability of development is between 0.5 and 2.5%, this suggests that sunflower is unlikely to sustain a viable population and *L. setosipennis* will not cause any major damage to any sunflower cultivars tested in the current study. In addition to this, *L. setosipennis* has never been recorded as a pest or even known by entomologists in South America [21].

There is no doubt that parthenium is a major problem in Pakistan [6–14] and control of this weed is imperative. Chemical control, although effective in Pakistan [28], is not a long-term solution, as it is extremely expensive and known to require several successive applications [29]. Biological control is known to offer a sustainable and effective option in several cases, especially in low and middle income countries [30], and *L. setosipennis* is no exception for Pakistan. In Pakistan, two biological control agents against parthenium are present, *Z. bicolorata* and *P. abrupta* var. *partheniicola* [20]; however, their impact is not considered sufficient, and additional agents are required. In Australia, where biological control is considered successful, nine different agents were required to be released [31]. Building on this success, additional agents should be studied for release in Pakistan in the future.

## 8. Conclusions

To reflect changes in the paper, as well as remove any reference to data in an unpublished report, the conclusions required changes, corrected paragraph:

The only known host of *L. setosipennis* in its native range of Argentina is parthenium [21]. The Australian research programme assessed 68 non-target plant species, including 18 members of the Asteraceae (including six sunflower cultivars) as well as commercially cultivated members from another 25 families [21]. In South Africa, *L. setosipennis* was tested on 38 native and economically important non-target Asteraceae species and 13 *H. annuus cultivars* [27], while in Ethiopia, it was evaluated on 31 plant species [22]. All countries where it has been released concluded that *L. setosipennis* is host specific and a safe biological control agent for use against parthenium, leading to approval for release by all regulatory authorities after a thorough review of submitted risk assessments. The quarantine laboratory assessments of the host range of *L. setosipennis* conducted in Pakistan are in line with these findings and in combination with evidence of the weevil’s native field host range in South America and introduced field host range in Australia and Ethiopia indicate that it is highly unlikely that *L. setosipennis* will cause any damage to any plant, native or cultivated, other than parthenium, in Pakistan.

## 9. Acknowledgments

Acknowledgments reflecting the contributions of personnel of the ARC-PHP have been added, corrected text:

We extend our gratitude to Lorraine Strathie of the ARC-PHP, Cedara, South Africa, for guidance during the experiments and support in the biological control of parthenium. We are grateful to Angela Bownes of the ARC-PHP, Cedara, South Africa, for conducting no-choice tests on sunflower cultivars. We are grateful to the ARC-PHP, Cedara, for training researchers from Pakistan in South Africa and providing a starter colony of *Listronotus setosipennis*, facilitating this study. The authors would like to thank Daud Hussain Anjum, research associate for his contribution and dedication in the maintenance of the cultures in quarantine as well as data collection in Pakistan. We highly appreciate the facilitation of the Department of Plant Protection and Pakistan Agricultural Research Council, Pakistan in establishing the quarantine laboratory at CABI Pakistan Centre and granting permission to import *L. setosipennis* into Pakistan to undergo host range testing in quarantine. We extend our gratitude to the Pakistan National Insect Museum (NIM), National Agriculture Research Centre (NARC), Islamabad, for lodging specimens of *Listronotus setosipennis* under voucher specimen number C/3666.

## 10. Supplementary Materials

A reference to a plant species tested in South Africa needed to be removed. Table legend is as follows:

**Supplementary Materials:** The following are available online at https://www.mdpi.com/article/10.3390/insects12050463/s1, Table S1: The test plant species from Australia and Ethiopia that have already been tested with *Listronotus setosipennis* in each respective country prior to release. Each country found no evidence for the potential of *L. setosipennis* to have non-target impacts on either native or economically important crop species.

The authors apologize for any inconvenience caused and state that the scientific conclusions are ultimately unaffected, despite the significant reworking of the paper. The original article has been updated.

## Figures and Tables

**Table 1:** The no-choice oviposition tests with *Listronotus setosipennis* against indigenous and economically important Asteraceae plant species in Pakistan between 2019 and 2020.

FAMILY	Common Name	No. Valid Replicates	No. of Eggs Mean ± SE
Tribe			
*Species*			
**ASTERACEAE**			
**Heliantheae**			
*Parthenium hysterophorus*	Gajar booti	25	185.5 ± 15.2
*Cosmos bipinnatus* ^E^	Cosmos	4	0 ± 0
*Eclipta prostrata* ^I^	Bhangra weed	4	0 ± 0
*Helianthus annuus* (S278) * ^E^	Sunflower	5	5.4 ± 1.7
*Helianthus annuus* (ParSun-3) * ^E^	Sunflower	5	16.2 ± 5.3
*Helianthus annuus* (SF-0054) * ^E^	Sunflower	5	9.6 ± 2.7
*Helianthus annuus* (KQS-FSH-1) * ^E^	Sunflower	5	9.8 ± 5.0
*Helianthus annuus* (S-3950) * ^E^	Sunflower	5	18.0 ± 7.9
*Helianthus annuus* (FMC-2) * ^E^	Sunflower	5	5.4 ± 2.0
*Helianthus annuus* (S-2216) * ^E^	Sunflower	5	12.8 ± 5.7
*Helianthus annuus* (ESNH-013) * ^E^	Sunflower	5	20.6 ± 6.3
*Helianthus annuus* (HySun-33) * ^E^	Sunflower	5	5.6 ± 4.5
*Helianthus annuus* (SX-4045) * ^E^	Sunflower	5	5.6 ± 3.7
*Rudbeckia laciniata* ^E^	Black-eyed Susan	4	0 ± 0
*Zinnia elegans* ^E^	Zinnia	4	0 ± 0
**Anthemideae**			
*Dendranthema indica* ^E^	Gul-e-Daudi	4	0 ± 0
**Astereae**			
*Callistephus chinensis* ^E^	Aster	4	0 ± 0
**Calenduleae**			
*Calendula officinalis* ^E^	Pot marigold	4	0 ± 0
**Cicorieae**			
*Lactuca sativa* ^E^	Lettuce	4	0 ± 0
**Coreopsideae**			
*Dahlia pinnata* ^E^	Dahlia	4	0 ± 0
*Bidens bipinnata* ^I^	Bidens	4	0 ± 0
**Cynareae**			
*Carthamus tinctorius* ^E^	Safflower	4	0 ± 0
**Tageteae**			
*Tagetes erecta* ^E^	Gul-e-Ashrafii	4	0 ± 0

^E^ indicates economically important species or cultivars in Pakistan. ^I^ indicates indigenous species in Pakistan. * Tests conducted in South Africa by ARC-PHP.
