# Supplementary material for: The Host Range and Risk Assessment of the Stem-Boring Weevil, Listronotus setosipennis (Coleoptera: Curculionidae) Proposed for the Biological Control of Parthenium hysterophorus (Asteraceae) in Pakistan
Source: Insects. 2021 May 17;12(5):463. doi: 10.3390/insects12050463 (PMC8156606; doi:10.3390/insects12050463)
Supplement: Supplementary file 1 [file insects-12-00463-s001.zip › insects-1202888-supplementary.pdf]

Supplementary Table S1. The test plant species from Australia, South Africa and Ethiopia that have already been tested with *Listronotus setosipennis* in each respective country prior to release. Each country found no evidence for the potential of *L. setosipennis* to have non-target impacts on either native or economically important crop species.

Family	Tribe	Species (Cultivar)	Country where tested
Asteraceae	Anthemideae	*Argyranthemum frutescens*	South Africa
Asteraceae	Anthemideae	*Artemisia afra*	South Africa
Asteraceae	Anthemideae	*Chrysanthemum sinense*	Australia
Asteraceae	Anthemideae	*Dendrathema x. grandiflorum*	South Africa
Asteraceae	Anthemideae	*Schistostephium flabelliforme*	South Africa
Asteraceae	Anthemideae	*Schistostephium heptalobum*	South Africa
Asteraceae	Arctotideae	*Arctotis arctotoides*	South Africa
Asteraceae	Astereae	*Aster novi-belgii*	South Africa
Asteraceae	Astereae	*Baccharis halimifolia*	Australia
Asteraceae	Astereae	*Conyza bonariensis*	Ethiopia
Asteraceae	Astereae	*Microglossa mespilifolia*	South Africa
Asteraceae	Calenduleae	*Dimorphotheca caulescens*	South Africa
Asteraceae	Calenduleae	*Garuleum sonchifolium*	South Africa
Asteraceae	Calenduleae	*Osteospermum muricatum* subsp. *Muricatum*	South Africa
Asteraceae	Cichorieae	*Cichorium intybus*	Australia, South Africa
Asteraceae	Cichorieae	*Lactuca sativa*	Australia, Ethiopia, South Africa
Asteraceae	Coreopsideae	*Bidens pilosa*	Australia
Asteraceae	Coreopsideae	*Bidens schimperi*	South Africa
Asteraceae	Coreopsideae	*Coreopsis grandiflora*	South Africa
Asteraceae	Coreopsideae	*Dahlia* sp.	Australia
Asteraceae	Coreopsideae	*Dahlia rosea*	South Africa
Asteraceae	Cynareae	*Carthamus tinctorius*	Australia, Ethiopia
Asteraceae	Eupatorieae	*Adenostemma caffrum*	South Africa
Asteraceae	Eupatorieae	*Adenostemma viscosum*	South Africa
Asteraceae	Eupatorieae	*Ageratina riparia*	Australia
Asteraceae	Gnaphalieae	*Athrixia phylicoides*	South Africa
Asteraceae	Gnaphalieae	*Callilepis laureola*	South Africa
Asteraceae	Gnaphalieae	*Cassinia laevis*	Australia
Asteraceae	Heliantheae	*Acanthospermum hispidum*	Australia
Asteraceae	Heliantheae	*Ambrosia tenuifolia*	Australia
Asteraceae	Heliantheae	*Bidens ghedoensis*	Ethiopia
Asteraceae	Heliantheae	*Bidens pilosa*	Ethiopia
Asteraceae	Heliantheae	*Bidens pachyloma*	Ethiopia
Asteraceae	Heliantheae	*Blainvillea gayana*	South Africa
Asteraceae	Heliantheae	*Cosmos* sp.	Australia
Asteraceae	Heliantheae	*Eclipta alba*	Australia

Family	Tribe	Species (Cultivar)	Country where tested
Asteraceae	Heliantheae	*Galinsoga parviflora*	Ethiopia
Asteraceae	Heliantheae	*Helianthus annuus* (R. Black)	Ethiopia
Asteraceae	Heliantheae	*Helianthus annuus* (Oissa)	Ethiopia
Asteraceae	Heliantheae	*Helianthus annuus* (Hysun 21)	Australia
Asteraceae	Heliantheae	*Helianthus annuus* (AFG 271)	South Africa
Asteraceae	Heliantheae	*Helianthus annuus* (AGSUN 5383)	South Africa
Asteraceae	Heliantheae	*Helianthus annuus* (AGSUN 5551)	South Africa
Asteraceae	Heliantheae	*Helianthus annuus* (AGSUN 8251)	South Africa
Asteraceae	Heliantheae	*Helianthus annuus* (DK 4040)	South Africa
Asteraceae	Heliantheae	*Helianthus annuus* (DKF 68-22)	South Africa
Asteraceae	Heliantheae	*Helianthus annuus* (Hysun 333)	South Africa
Asteraceae	Heliantheae	*Helianthus annuus* (PAN 7033)	South Africa
Asteraceae	Heliantheae	*Helianthus annuus* (PAN 7048)	South Africa
Asteraceae	Heliantheae	*Helianthus annuus* (PAN 7049)	South Africa
Asteraceae	Heliantheae	*Helianthus annuus* (PAN 7050)	South Africa
Asteraceae	Heliantheae	*Helianthus annuus* (PAN 7351)	South Africa
Asteraceae	Heliantheae	*Helianthus annuus* (Sirena)	South Africa
Asteraceae	Heliantheae	*Helianthus annuus* (Polestar)	Australia
Asteraceae	Heliantheae	*Helianthus annuus* (Sunbred 27)	Australia
Asteraceae	Heliantheae	*Helianthus annuus* (Suncross)	Australia
Asteraceae	Heliantheae	*Helianthus annuus* (Sungold)	Australia
Asteraceae	Heliantheae	*Helianthus annuus* (Yates Sunbred)	Australia
Asteraceae	Heliantheae	*Helianthus tuberosus*	South Africa
Asteraceae	Heliantheae	*Melanthera scandens* subsp. *dregei*	South Africa
Asteraceae	Heliantheae	*Parthenium argentatum*	Australia
Asteraceae	Heliantheae	*Siegesbeckia orientalis*	Australia
Asteraceae	Heliantheae	*Spilanthes mauritiana*	South Africa
Asteraceae	Heliantheae	*Wedelia natalensis*	South Africa
Asteraceae	Heliantheae	*Xanthium strumarium*	South Africa
Asteraceae	Heliantheae	*Zinnia angustifolia*	South Africa
Asteraceae	Heliantheae	*Zinnia* sp.	Australia
Asteraceae	Millerieae	*Guizotia abyssinica* (ESTE)	Ethiopia
Asteraceae	Millerieae	*Guizotia abyssinica* (Fogera)	Ethiopia
Asteraceae	Millerieae	*Guizotia abyssinica* (Kuyu)	Ethiopia
Asteraceae	Millerieae	*Guizotia abyssinica* (Kuyu)	South Africa
Asteraceae	Millerieae	*Guizotia abyssinica* (Local)	Ethiopia
Asteraceae	Millerieae	*Guizotia abyssinica* (Shambu)	Ethiopia
Asteraceae	Millerieae	*Guizotia abyssinica* (Shambu)	South Africa
Asteraceae	Millerieae	*Guizotia abyssinica* (Ghinchi)	Ethiopia
Asteraceae	Millerieae	*Guizotia scabra*	Ethiopia

Family	Tribe	Species (Cultivar)	Country where tested
Asteraceae	Neurolaeneae	*Galinsoga parviflora*	Australia
Asteraceae	Senecioneae	*Senecio* ‘shrubby’ *tamoides*	South Africa
Asteraceae	Senecioneae	*Senecio brachypodus*	South Africa
Asteraceae	Senecioneae	*Senecio deltoideus*	South Africa
Asteraceae	Senecioneae	*Senecio microglossus*	South Africa
Asteraceae	Senecioneae	*Senecio tamoides*	South Africa
Asteraceae	Senecioneae	*Solanecio angulatus*	South Africa
Asteraceae	Tageteae	*Flaveria trinervia*	Ethiopia
Asteraceae	Tageteae	*Tagetes minuta*	Ethiopia
Asteraceae	Tageteae	*Tagetes patula*	South Africa
Asteraceae	Vernonieae	*Ethulia conyzoides* subsp. *kraussii*	South Africa
Asteraceae	Vernonieae	*Vernonia galamensis*	Ethiopia
Asteraceae	Vernonieae	*Vernonia natalensis*	South Africa
Anacardiaceae		*Mangifera indica*	Australia
Annonaceae		*Annona reticulata*	Australia
Apiaceae		*Apium graveolens*	Australia
Apiaceae		*Daucus carota*	Australia
Apiaceae		*Petroselinum crispum*	Australia
Brassicaceae		*Brassica carinata*	Ethiopia
Brassicaceae		*Brassica oleracea*	Australia, Ethiopia
Brassicaceae		*Brassica rapa*	Australia
Bromeliaceae		*Ananas comosus*	Australia
Caricaceae		*Carica papaya*	Australia
Chenopodiaceae		*Beta vulgaris*	Australia
Convolvulaceae		*Ipomoea batata*	Australia
Cucurbitaceae		*Cucumis sativus*	Australia
Cucurbitaceae		*Cucurbita maxima*	Australia
Fabaceae		*Arachis hypogaea*	Australia
Fabaceae		*Cicer arietinum*	Ethiopia
Fabaceae		*Glycine max*	Australia
Fabaceae		*Lathyrus sativus*	Ethiopia
Fabaceae		*Lens cultinaris*	Ethiopia
Fabaceae		*Macroptilium atropupureum*	Australia
Fabaceae		*Medicago sativa*	Australia
Fabaceae		*Phaseolus vulgaris*	Australia
Fabaceae		*Pisum sativum*	Ethiopia
Fabaceae		*Vicia faba*	Ethiopia
Lauraceae		*Persea americana*	Australia
Liliaceae		*Allium cepa*	Australia
Linaceae		*Linum usitatissimum*	Australia

Family	Tribe	Species (Cultivar)	Country where tested
Malvaceae		*Corchorus olitorius*	Ethiopia
Malvaceae		*Gossypium hirsutum*	Ethiopia
Malvaceae		*Gossypium* sp.	Australia
Musaceae		*Eucalyptus grandis*	Australia
Musaceae		*Melaleuca armillaris*	Australia
Passifloraceae		*Passiflora edulis*	Australia
Pedaliaceae		*Sesamum indicum*	Ethiopia
Pinaceae		*Pinus elliotti*	Australia
Poaceae		*Chloris gayana*	Australia
Poaceae		*Dicanthium sericeum*	Australia
Poaceae		*Eragrostis tef*	Ethiopia
Poaceae		*Hordeum vulgare*	Australia
Poaceae		*Lolium multiflorum*	Australia, South Africa
Poaceae		*Lolium perenne*	Australia
Poaceae		*Saccharum officinarum*	Australia
Poaceae		*Sorghum bicolor*	Ethiopia
Poaceae		*Sorghum vulgare*	Australia
Poaceae		*Triticum aestivum*	Australia, Ethiopia
Poaceae		*Zea mays*	Australia, Ethiopia
Proteaceae		*Macadamia integrifolia*	Australia
Rosaceae		*Fragaria vesca*	Australia
Rosaceae		*Malus sylvestris*	Australia
Rosaceae		*Prunus domesticus*	Australia
Rosaceae		*Prunus persica*	Australia
Rosaceae		*Pyrus communis*	Australia
Rosaceae		*Rosa* sp.	Australia
Rubiaceae		*Coffea arabica*	Australia
Rutaceae		*Citrus sinense*	Australia
Solanaceae		*Capsicum annum*	Australia, Ethiopia
Solanaceae		*Lycopersicon esculentum*	Australia
Solanaceae		*Nicotiana tabacum*	Australia
Solanaceae		*Solanum lycopersicum*	Ethiopia
Solanaceae		*Solanum tuberosum*	Australia, Ethiopia
Vitaceae		*Vitis vinifera*	Australia
Zingiberaceae		*Zingiber officinale*	Australia
